# Conceptualization, use, and outcomes associated with compassion in the care of youth with childhood-onset disabilities: a scoping review

**DOI:** 10.3389/fpsyg.2024.1365205

**Published:** 2024-06-07

**Authors:** Eleni M. Patsakos, Stuti Patel, Robert Simpson, Michelle L. A. Nelson, Melanie Penner, Laure Perrier, Mark T. Bayley, Sarah E. P. Munce

**Affiliations:** ^1^Rehabilitation Sciences Institute, University of Toronto, Toronto, ON, Canada; ^2^KITE Research Institute - Toronto Rehabilitation Institute, University Health Network, Toronto, ON, Canada; ^3^Institute of Health and Wellbeing, University of Glasgow, Glasgow, United Kingdom; ^4^Division of Physical Medicine and Rehabilitation, Temerty Faculty of Medicine, University of Toronto, Toronto, ON, Canada; ^5^Lunenfeld-Tanenbaum Research Institute, Toronto, ON, Canada; ^6^Institute of Health Policy, Management and Evaluation, University of Toronto, Toronto, ON, Canada; ^7^Autism Research Centre, Holland Bloorview Kids Rehabilitation Hospital, Toronto, ON, Canada; ^8^Department of Paediatrics, Faculty of Medicine, University of Toronto, Toronto, ON, Canada

**Keywords:** compassion, childhood-onset disabilities, scoping review, youth & adolescence, self-compassion, childhood disability, disability, caregiver

## Abstract

**Introduction:**

To examine the scope of existing literature on the conceptualization, use, and outcomes associated with compassion in the care of youth with childhood-onset disabilities.

**Methods:**

A protocol was developed based on the Joanna Briggs Institute (JBI) scoping review method. MEDLINE, EMBASE, PsycINFO, Cochrane Central Register of Controlled Trials, and EBSCOhost CINAHL, were searched.

**Results:**

Eight studies were selected for inclusion; four used quantitative methodology, and four used qualitative methods. Compassion was not defined *a priori* or *a posteriori* in any of the included studies. The concept of self-compassion was explicitly defined only for parents of youth with childhood-onset disabilities in three studies *a priori*. The most reported outcome measure was self-compassion in parents of youth with childhood-onset disabilities. Self-compassion among parents was associated with greater quality of life and resiliency and lower stress, depression, shame and guilt.

**Discussion:**

There is limited evidence on the conceptualization, use, and outcomes associated with compassion among youth with childhood-onset disabilities. Self-compassion may be an effective internal coping process among parents of youth with childhood-onset disabilities. Further research is required to understand the meaning of compassion to youth with childhood-onset disabilities, their parents and caregivers.

**Systematic review registration:**

https://doi.org/10.17605/OSF.IO/2GRB4.

## Highlights

- The conceptualization of compassion or self-compassion among youth with childhood-onset disabilities was not discussed.
- Compassion was not defined *a priori* or *a posteriori* in any of the included studies.
- Self-compassion was conceptualized by established definitions only for parents of youth with childhood-onset disabilities.
- Several studies liberally used the terms ‘compassion’ and ‘compassionate care’.
- The Mindfulness-Based Positive Behavior Support program was significantly better at enhancing compassion satisfaction and reducing caregiver-perceived psychological stress.

## Introduction

Compassion is deeply ingrained in numerous religious and spiritual traditions worldwide. Over the last 30 years, compassion has received increasing attention from the scientific community, recognizing its benefits and positive associations with quality of life, clinical outcomes, and satisfaction with healthcare services (Sinclair et al., 2016; Strauss et al., 2016; Malenfant et al., 2022). Many philosophers have stated that compassion is a “morally praiseworthy emotion” (Cameron and Rapier, 2017). Echoing this sentiment, the Dalai Lama poignantly stated, “Love and compassion are necessities, not luxuries. Without them, humanity cannot survive” (Cutler and Lama, 2011). Deriving from the Latin word ‘compati,’ meaning ‘to suffer with,’ a range of definitions have been developed by scholars from both Western and Buddhist psychological perspectives (Neff, 2003a; Kanov et al., 2004; Gilbert, 2009; Goetz et al., 2010; Pommier, 2010; Feldman and Kuyken, 2011; Strauss et al., 2016). To consolidate the past conceptualizations and definitions of compassion, Strauss et al. (2016) have proposed a new definition of compassion:

“a cognitive, affective and behavioral process consisting of the following five elements that refer to both self and other-compassion: (1) recognizing suffering; (2) understanding the universality of suffering in human experience; (3) feeling empathy for the person suffering and connecting with the distress (emotional resonance); (4) tolerating uncomfortable feelings aroused in response to the suffering person (e.g., distress, anger, fear) so remaining open to and accepting of the person suffering, and (5) motivation of act/acting to alleviate suffering” (Strauss et al., 2016).

Compassion is often included in the competency frameworks and codes of ethics of professional healthcare organizations and is a crucial component of providing quality healthcare (Sinclair et al., 2016; Malenfant et al., 2022; Simpson et al., 2022). Several physician behaviors identified by patients as important to providing compassion within healthcare interactions include: (1) listening and paying attention to the patient; (2) respecting preferences (i.e., physicians are culturally competent, respect the patient’s preferences and ensure patients are actively involved in healthcare decisions); (3) continuity of care (i.e., long-term management of chronic conditions with a physician); (4) genuine understanding (i.e., seeking to understand the patient’s situation and symptoms to attempt to identify a cause); (5) following-up on patient test results; (6) body language (i.e., not speaking over a patient, physician moves their chair to face patient, and explaining diagnosis in a manner the patient will understand); and (7) counseling and advocacy (i.e., being non-judgmental toward concerns related to mental health, chronic illness and diagnoses with somatic complaints, showing sensitivity, and providing counseling and advocacy related to these concerns) (Baguley et al., 2022). Providing compassion within healthcare interactions has been shown to improve patient outcomes (i.e., quality of life, satisfaction with the quality of healthcare received), improve their relationship with healthcare providers, lower intensive care utilization, increase autonomy and faster recovery times (Van der Cingel, 2011; Sharp et al., 2016; Sinclair et al., 2016; Baguley et al., 2022; Malenfant et al., 2022). Further, compassion positively impacts several clinician outcomes by improving overall workplace well-being, job satisfaction and retention rates (Sinclair et al., 2022).

Modern theorists have stated that compassion is “reproductively advantageous” and has been a component of the “caregiving system” to protect and nurture children (Goetz et al., 2010; Strauss et al., 2016). The notion of compassion has further evolved to focus on protecting others “beyond one’s immediate kinship group” (Strauss et al., 2016). The concept of compassion developed as an “affective state” focused on improving the individuals who suffer for three reasons: it enhances the “welfare of vulnerable offspring,” it is a desirable trait during the mate selection process and allows for the cooperation of individuals who are non-kin (Goetz et al., 2010).

A related concept, self-compassion, is a multifaceted construct that involves “being open to one’s own suffering and to heal oneself with kindness” (Neff, 2003a). It involves recognizing one’s own experiences within the broader context of the universal human experience, understanding that suffering, failure, and shortcomings are inherent aspects of being human, and acknowledging that all individuals, including oneself, deserve compassion (Neff, 2003a). Neff (2023) notes that contrary to many psychological theories, individuals frequently exhibit a lack of kindness toward themselves compared to how they would treat those they care about or strangers, which may arise from a fear of appearing egotistical, self-indulgent, or self-centered.

Self-compassion includes three primary components: (a) extending self-kindness to oneself instead of self-judgment and self-criticism, (b) recognition of common humanity (i.e., viewing “one’s experiences as part of the broader human experience” (Neff, 2003a) rather than as isolating), and (c) mindfulness rather than over-identification (Neff, 2003a). Self-kindness refers to having a caring and understanding attitude toward oneself, rather than adopting a harshly critical or judgmental stance. Common humanity entails acknowledging that all individuals have imperfections and experience failures. Mindfulness, considered to be the “pillar” of self-compassion, entails maintaining a clear and balanced awareness of one’s “present-moment experiences” (Neff, 2009) to avoid ruminating on the negative aspects of oneself or one’s life. Mindfulness increases self-understanding and lessens self-criticism (Neff, 2003b) thereby enhancing self-kindness (Neff, 2003a). It provides the space for individuals to ask themselves, “How can I care for myself right now?” These elements blend and interact synergistically to create a “self-compassionate frame of mind” (Neff, 2003a). Further, self-compassion has been associated with several positive outcomes, including increased life satisfaction, happiness, connectedness to others, and optimism, and inversely correlated with mental health disorders such as anxiety, depression, self-criticism and stress in both clinical and community populations (Neff et al., 2005; Neff, 2009; Hollis-Walker and Colosimo, 2011; Finlay-Jones et al., 2015). Psychological research has shown several prosocial outcomes when individuals feel compassion for others including volunteering to help others.

Previous studies examining compassion in pediatric populations have focused on pediatric oncology healthcare settings, recognizing compassion as a component of the overall healthcare experience and providing effective clinical communication and shared decision-making (Rider and Perrin, 2002; Kuo et al., 2012; Brooten et al., 2013; Orioles et al., 2013; Sinclair et al., 2016, 2020). The insights gleaned from pediatric oncology could be transferrable to other populations. Several facilitators of compassion within pediatric oncology include continuity of care (i.e., the process by which pediatric patients and healthcare providers manage patient care to improve care), communication about care (i.e., the tone, language, style and cultural sensitivity of the information being provided to the patient and their family) and coordination of care (i.e., coordination of patient healthcare activities and sharing this information with patients, family caregivers and allied health professionals to provide optimal patient-centered care) (Sinclair et al., 2020). Self-compassion has been reported as a potential coping resource for parents and caregivers of youth with childhood-onset disabilities and is linked to lower levels of stress and depression (Robinson et al., 2018). While previous reviews on compassion have focused on pediatric oncology patients (Sinclair et al., 2020), no reviews have focused on the concept of compassion in other pediatric populations, including youth with childhood-onset disabilities.

Childhood-onset disabilities are defined as a “group of congenital or acquired chronic conditions that originate during pregnancy or infancy and comprise impairments in physical, intellectual, and/or behavioral function” (Peterson and Hurvitz, 2021). These conditions and disorders begin in the early stages of development (conception, birth, infancy, childhood) and are characterized by motor, cognitive, behavioral, linguistic, and socio-emotional challenges (Brehaut et al., 2004; Andrews et al., 2015). Worldwide, approximately 15 percent of the pediatric population is diagnosed with a childhood-onset disability. Examples of childhood-onset disability include autism spectrum disorder (ASD), cerebral palsy, intellectual and developmental disabilities, traumatic brain injury (TBI), acquired brain injury (ABI), and fetal alcohol spectrum disorder (FASD). Youth with childhood-onset disabilities often experience complex medical, social, emotional and educational support needs that impact parental and caregiver well-being and family function (Miller et al., 2022).

Self-compassion in youth is positively correlated with social connectedness, secure attachment and adaptive coping and negatively associated with fearful and preoccupied attachment (Bluth and Clepper-Faith, 2023). This finding is significant because forming healthy peer relationships and maintaining stable social networks plays a crucial role in emotional well-being during adolescence (Bluth and Clepper-Faith, 2023). Further, learning to manage stressful situations in a healthy manner is crucial for youth as they transition into adulthood (Bluth and Clepper-Faith, 2023). Developing the ability to cope effectively with stress is important for healthy adolescent development (Bluth and Clepper-Faith, 2023). Understanding the meaning of compassion and self-compassion for youth with childhood-onset disabilities and their family caregivers will assist in developing novel interventions that can improve health outcomes and fully address the needs of the youth and their caregivers. To our knowledge, the literature surrounding the conceptualization, use and outcomes of compassion in the care of youth with childhood-onset disabilities has not been synthesized. The objective of this review is to describe the existing literature on the conceptualization (i.e., *a priori* or posteriori definitions of compassion and self-compassion), use (i.e., the application of compassion and self-compassion), and outcomes associated with compassion in the care of youth with childhood-onset disabilities. The following research questions are examined: How are compassion conceptualized and used in the care of youth with childhood-onset disabilities? What are the outcomes associated with compassion in the care of youth with childhood-onset disabilities?

## Method

This scoping review was conducted in accordance with the Joanna Briggs Institute (JBI) methodological guidance for scoping reviews (Tricco et al., 2018). The Preferred Reporting Items for Systematic Reviews and Meta-Analyses extension for Scoping Reviews (PRISMA-ScR) (Tricco et al., 2018) was used to guide the reporting of this review. The PRISMA-ScR checklist is provided in Supplementary Appendix S1.

A scoping review methodology was selected to determine the scope of the literature, identify and map the existing compassion literature within childhood-onset disability, clarify existing definitions of compassion and self-compassion, and identify existing knowledge gaps within this emerging field (Munn et al., 2018; Tricco et al., 2018).

### Protocol and registration

The protocol for this review was registered on the Open Science Framework Register on November 14, 2022 (Registration DOI: https://doi.org/10.17605/OSF.IO/2GRB4).

### Eligibility criteria

The eligibility criteria were developed using the PCC (population, concept, context) framework, one of three recommended by JBI (Tricco et al., 2018).

### P – Population

The review included youth with childhood-onset disabilities from 12 to 25 years of age, families, and healthcare professionals. Studies were selected for inclusion if the mean age of youth with childhood-onset disabilities was under ≤25 years. The age range of 12–25 has been previously used to synthesize literature on youth with childhood-onset disabilities (Levy et al., 2020). The following questions are addressed in this scoping review: How is compassion conceptualized in the care of youth with childhood-onset disabilities?

### C – Concept

The review focused on sources of evidence describing the conceptualization (i.e., *a priori* or posteriori definitions of compassion and self-compassion), use (i.e., the application of compassion and self-compassion), and outcomes associated with compassion.

### C – Context

Sources of evidence from any country, study setting, and time frame were eligible for inclusion.

### Year of publication, publication status and language

The review sought to identify peer-reviewed sources using qualitative, quantitative or mixed methods study designs published from database inception to June 22, 2022. The review was limited to only sources published in English. Dissertations, conference abstracts, letters to the editor and editorials were excluded.

### Search strategy

The literature search strategy was designed and conducted by an experienced information specialist (LP) with input from the research team. A comprehensive literature search was run in Ovid MEDLINE, Ovid MEDLINE Daily, Ovid MEDLINE In-Process & Other Non-Indexed Citations, Ovid MEDLINE Epub Ahead of Print, OVID EMBASE, OVID PsycINFO, Cochrane Central Register of Controlled Trials, and EBSCOhost CINAHL with medical subject headings and keywords related to compassion, empathy, youth, neurodevelopmental disability and childhood-onset disability using search symbols and Boolean operators. Relevant wildcard terms were included to account for spelling variations and plural terms. No year or language limitations were placed on the search. The search was run on June 22, 2022. The MEDLINE search strategy can be found in Supplementary Appendix S2.

### Evidence screening and selection

All identified citations were exported to the reference manager, EndNote 20 (Clarivate Analytics, 2020), and duplicates were removed. A standardized screening form to determine study eligibility was developed and independently pilot-tested using the same 15 randomly selected titles and abstracts by two reviewers (EP, SP). Following one pilot session, an inter-rater agreement of ≥ 80% was reached on the inclusion/exclusion status of the articles. Level I and II screening were conducted using Covidence. Titles and abstracts were independently screened (i.e., level I screening) by two reviewers (EP, SM, SP). Two independent reviewers completed full-text screening of potentially relevant articles (i.e., level II screening) (EP, SP). The interrater agreement following pilot and full-text screening indicated ‘substantial agreement’ with a Cohen’s Kappa statistic of *κ* = 0.90 and *κ* = 0.92, respectively (McHugh, 2012).

### Data extraction

A standardized data extraction form was pilot-tested by two independent reviewers (EP, SP) on a sample of three studies in Covidence to ensure all relevant data was extracted. Following the pilot extraction of the three studies, agreement was reached by the reviewers on what data should be extracted. The data extraction form was modified using an iterative process (Tricco et al., 2018). Data extraction was conducted in duplicate within Covidence. The following data were extracted: study author(s), year of publication, country, study setting, study design, methodology, population description (i.e., youth with childhood-onset disabilities, caregivers, parents, etc.), sample size, participant characteristics (i.e., sex, mean age, etc.), intervention/comparator (if applicable), tools used to measure compassion, data collection time points, findings related to the conceptualization, use and outcomes associated with compassion in the care of youth with childhood-onset disabilities.

**Table 1:** Study characteristics.

Study	Setting/country	Study design	Population, *n*	Intervention	Intervention adherence	Tools to measure compassion	Data collection points
Biddle et al. (2020)	Child health research centre, Australia	Quantitative; cross-sectional survey	Caregivers of children, adolescents and young adults diagnosed with FASD, *n* = 175	N/A	N/A	DASS-2, SCS-SF, SSGS	Single time point
Conti (2015)	Interactive Autism Network, United States	Quantitative; cross-sectional survey	Study I Parents, *n* = 76 Mothers: 97.4% Fathers: 2.6%* Study II Parents, *n* = 129 Mothers: 100%	N/A	N/A	SWLS, MLQ, KFLS, PGQ	Single time point
Iannuzzi et al. (2022)	Academic medical center, United States	Qualitative; semi-structured interviews	Parents of individuals with ASD, *n* = 21 Neurotypical adolescents and young adult siblings of individuals with ASD, *n* = 20	N/A	N/A	N/A	Single time point
Ivins-Lukse and Lee (2021)	Community organization, United States	Quantitative; cross-sectional survey	Parents of transition-age youth with IDD, *n* = 100	N/A	N/A	DCFS, Self-Compassion Scale, Satisfaction with life Scale CES-D-10**	Single time point
Moore et al. (2015)	Two Level 1 trauma centers, United States	Qualitative; semi-structured interviews	Mothers of children with TBI who had an acute hospital stay, *n* = 15	N/A	N/A	N/A	Single time point
O’Reilly (2017)	Residential aged care facility, Australia	Qualitative; interviews	Paid carers, *n* = 15	N/A	N/A	N/A	Single time point
Robinson et al. (2018)	Academic medical center, Canada	Quantitative; cross-sectional survey	Parents of youth with IDD, *n* = 56	N/A	N/A	SCS-SF	Single time point
Singh et al. (2020)	Group home for adolescents and adults with ID and ASD; United States	Quantitative; three-arm cluster RCT with two active conditions and a control condition	Paid caregivers, *n* = 216 Mindfulness program, *n* = 72 Psychoeducational program, *n* = 72 Inservice training-as-usual program (control), *n* = 72	Mindfulness program Psychoeducational program Inservice training-as-usual program (control)	Attendance to all intervention programs was 100%	PSS-10, ProQOL BDI-II	Baseline, post-intervention (32-week implementation)

^*^Due to the small number of fathers, only mothers were included in the analysis. ^**^Other measures not included within the present study but were collected within the same survey: Burden Assessment Scale, Brief COPE Inventory, Connor-Davidson Resilience Scale, Family Quality of Life, Generalized Anxiety Disorder 7, Multidimensional Scale of Perceived Social Support, Parenting Sense of Competence, and Perceived Stress Scale.

ASD, autism spectrum disorder; BDI-II, Beck Depression Inventory-II; BRI-SF, Behavior Problems Inventory-Short Form; CES-D-10, Center for Epidemiologic Studies Short Depression Scale; DASS-21, Depression Anxiety Stress Scale; DCFS, Devaluation of Consumer Families Scale; FASD, fetal alcohol spectrum disorder; HF-ASD, high-functioning autism spectrum disorder; ID, intellectual disability; IDD, intellectual and developmental disabilities; IFS, 24-item Impact on Family Scale; IRI, Interpersonal Reactivity Index; KFLS, Kansas Family Life Satisfaction; MLQ, Meaning in Life Questionnaire; MSPSS, Multidimensional Scale of Perceived Social Support; N/A, not applicable; PGQ, Parenting Goal Questionnaire; ProQOL, Professional Quality of Life; PSI-SF, Parenting Stress Index: Short Form; PSOC, Parenting Sense of Competence Scale; PSS-10, Perceived Stress Scale; RCT, randomized controlled trial; RSRRS, Revised Social Readjustment Rating Scale; SCQ, Social Communication Questionnaire; SCS-SF, Self-Compassion Scale Short Form; SRM-SF, Sociomoral Reflection Measure: Short Form; SSGS, State Shame and Guilt Scale; SWLS, Satisfaction with Life Scale; TBI, traumatic brain injury; TD, typically developing.

### Risk of bias appraisal

Consistent with established scoping review methods, the risk of bias was not appraised on articles selected for inclusion (Tricco et al., 2018; Peters et al., 2020).

### Synthesis of results

Data were quantitatively summarized using frequency counts (e.g., concepts, location of studies) and qualitatively using descriptive analysis. A meta-analysis of quantitative data was not conducted as it is not typically performed within scoping reviews (Peters et al., 2020).

## Results

A total of 2048 records were identified from the literature database searches before deduplication. EMBASE, MEDLINE, CINAHL, PsycINFO, and Cochrane Central Register of Controlled Trials retrieved 815, 688, 318, 207 and 20 records, respectively. Following deduplication, 895 duplicate records were removed. A total of 1,153 records were available for title and abstract screening and 947 records were excluded. The full text of the remaining 206 studies was reviewed, and eight studies met all study inclusion criteria. The PRISMA flow diagram is outlined in Supplementary Appendix S3.

### Study characteristics

The included studies were conducted in Australia (O’Reilly, 2017; Biddle et al., 2020), Canada (Robinson et al., 2018) and the United States (Conti, 2015; Moore et al., 2015; Singh et al., 2020; Ivins-Lukse and Lee, 2021; Iannuzzi et al., 2022) and published between 2015 and 2022. Five studies used quantitative methodology (Conti, 2015; Robinson et al., 2018; Biddle et al., 2020; Singh et al., 2020; Ivins-Lukse and Lee, 2021); three used qualitative methodology (Moore et al., 2015; O’Reilly, 2017; Iannuzzi et al., 2022) (Table 1).

### Participant characteristics

The majority of included studies were comprised of parents of youth with childhood-onset disabilities (Conti, 2015; Moore et al., 2015; Robinson et al., 2018; Biddle et al., 2020; Ivins-Lukse and Lee, 2021; Iannuzzi et al., 2022). The remaining two studies included paid caregivers: one within a residential aged care facility (O’Reilly, 2017; Singh et al., 2020) and another within a group home for adolescents and adults with intellectual disabilities and ASD (Singh et al., 2020). Participant ethnicity was reported in four studies (Conti, 2015; Moore et al., 2015; Ivins-Lukse and Lee, 2021; Iannuzzi et al., 2022), with most participants identifying as “Caucasian.” Included studies focused on youth with ASD (Iannuzzi et al., 2022), ABI (O’Reilly, 2017), TBI (Moore et al., 2015), FASD (Biddle et al., 2020), with the remaining four studies focused on youth with different childhood-onset disability diagnoses, including ASD (Conti, 2015; Robinson et al., 2018; Singh et al., 2020; Ivins-Lukse and Lee, 2021), and intellectual and developmental disability (Robinson et al., 2018; Singh et al., 2020; Ivins-Lukse and Lee, 2021). The age of youth reported in the included studies was between 12 to 25 years of age. Participant sex was reported in all studies. Most study participants were female, with a percentage of females between 53.5 and 100%. Socioeconomic status was only reported in one study, reporting a familial income of over USD 70,000 (Ivins-Lukse and Lee, 2021). Education status was reported in two studies, with most participants completing high school (Conti, 2015) and technical training or post-school skills (Ivins-Lukse and Lee, 2021). Across studies, the sample size ranged from 8 to 216 participants (Table 2).

**Table 2:** Participant characteristics.

Study	Participant sex (%)	Mean age (SD)	Socio-economic status	Ethnicity	Employment status	Education status	Type of NDD of youth
Biddle et al. (2020)*	Total *n* = 175 Female: 96% Male: 3.5% Non-Binary: 0.6%	Median age: 50.06 (mean age N/R) Age of children, adolescents and young adults diagnosed with FASD: 12.03 (4.64)	N/R	N/R	Currently in paid work: 49.1% Unemployed: 41.1% Permanently unable to work: 9.7%	Post-school skills or technical training: 30.4% Advanced diploma or diploma: 19.6% Bachelor degree: 29.8% Graduate degree: 8.9% Postgraduate degree: 11.3%	FASD
Conti (2015)	Study I Female: 97.4% Male: 2.6% Study II Female: 100%	Study I Mothers: 44.1 (SD N/R) Fathers: N/R Study II Mothers: 41.3 (6.01) Fathers: N/R	N/R	Study I African American: *n* = 1 Asian American/Pacific Islander: *n* = 1 Caucasian: *n* = 72 “Other Race”: *n* = 2 Comparison sample African American: *n* = 5 Asian American/Pacific Islander: *n* = 2 Caucasian: *n* = 34 Latinas: *n* = 5 Study II Asian American: *n* = 2 Caucasian: *n* = 119 Latino: *n* = 5 Not reported: *n* = 3	N/R	High school: 57% 4-year college degree: 22% Graduate degree: 21%	High-functioning Autism/Asperger’s Syndrome: *n* = 39 Autistic Disorder: *n* = 48 PDD-NOS: *n* = 27 ASD: *n* = 15
Iannuzzi et al. (2022)	Total: *n* = 41 Parents Female: 90.5% Male: 9.5% Siblings Female: 75% Male: 25%	Parents Median age (range): 51 (43–68)** Siblings Median age (range): 17 (13–24)	N/R	Parents Asian: 9.5% Caucasian: 81% Hispanic/Latino: *4.8%* Other: 9.5% Siblings Asian: 5% Caucasian: 80% Hispanic/Latino: 25% Other: 15%	N/R	N/R	ASD
Ivins-Lukse and Lee (2021)	Total *n* = 100 Female: 64% Male: 37%	46.97 (6.63) Age of youth with IDD: 17.22 (3.06)	Under US$10,000: 2% US$10,000–19,999: 7% US$20,000–29,999: 9% US$30,000–39,999: 5% US$40,000–49,999: 7% US$50,000–59,999: 9% US$60,000–$69,999: 27% Over US$70,000: 33%	Caucasian: 72% Hispanic: 17% Black: 8% Native American: 3%	Employed: 57% Unemployed: 43%	Years of education *M* (SD): 15.03 (2.44)	ASD: *n* = 57 ID: *n* = 16 DD: *n* = 7 Multiple: *n* = 19
Moore et al. (2015)	Total: *n* = 15 Female: 100%	N/R	N/R	African American: 20% Asian: 7% Caucasian: 7% Hispanic: 53% Multiracial: 13%	N/R	N/R	TBI (*n* = 15) Severe TBI (GCS < 8): 20% Moderate TBI (GCS 9–12): 20% Mild TBI: 60%
O’Reilly (2017)	Total: *n* = 15 Female: 53.3% Male: 46.7%	Range: 21–65*** Age of youth with ABI: N/R	N/R	N/R	Bachelor of Nursing: 40% Diploma of Nursing: 13.3% Nursing Assistant: 33.3% Non-Nursing Ancillary: 13.4%	N/R	ABI
Robinson et al. (2018)	Total: *n* = 56 Female: 70% Male: 30%	56.5 (8.8) Age of youth: 23.0 (5.9)	N/R	N/R	N/R	N/R	ASD: 41.1% Genetic syndrome: 14.2% Psychiatric disorder: 10.7%
Singh et al. (2020)	Total: *n* = 216 Mindfulness program Female: 58.3% Male: 41.7% Psychoeducational program Female: 52.8% Male: 47.2% Inservice training-as-usual program (control) Female: 54.2% Male: 45.8%	Mindfulness program: 40.68 Psychoeducational program: 37.89 Inservice training-as-usual program (control): 39. 61	N/R	N/R	N/R	N/R	ID ASD

^*^Participant gender provided. ^**^Missing *n* = 1. ^***^One participant chose to withhold his/her age.

ABI, acquired brain injury; ASD, autism spectrum disorder; DD, developmental disability; FASD, fetal alcohol spectrum disorder; HF-ASD, high-functioning autism spectrum disorder; ID, intellectual disability; IDD, intellectual and developmental disabilities; NDD, neurodevelopmental disability; N/R, not reported; PDD-NOS, Pervasive Developmental Disorder-Not Otherwise Specified.

### Conceptualization of compassion in the care of youth with childhood-onset disabilities

Compassion as a concept was not explicitly defined in any of the included studies. However, self-compassion was explicitly defined for parents of youth with childhood-onset disabilities in three studies *a priori* (Robinson et al., 2018; Biddle et al., 2020; Ivins-Lukse and Lee, 2021). Ivins and Lukse (2021) defined self-compassion as representing a form of emotion-based coping that specifically involves how an individual relates to themselves when facing difficult experiences. Biddle et al. (2020) defined self-compassion as the recognition and desire to alleviate one’s suffering with self-kindness and non-judgment. They described it as being associated with several positive outcomes (e.g., greater psychological health, reduced isolation, and improved well-being). Similarly, Robinson et al. (2018) described self-compassion as “being touched by and open to one’s own suffering, not avoiding or disconnecting from it, generating the desire to alleviate one’s suffering and to heal oneself with kindness.” Self-compassion was also described as “offering nonjudgmental understanding to one’s pain, inadequacies and failures, so that one’s experience is seen as part of the larger human experience.” The conceptualization of compassion or self-compassion among youth with childhood-onset disabilities was not discussed in any of the included studies.

### Use of compassion in the care of youth with childhood-onset disabilities

Three studies investigated the use of compassion among paid carers toward youth with ABI, intellectual disability and ASD (Moore et al., 2015; O’Reilly, 2017; Singh et al., 2020).

Only one interventional study met the scoping review inclusion criteria (Singh et al., 2020). Singh et al. (2020) randomized paid caregivers for individuals with intellectual disabilities or ASD in three experimental programs: (1) mindfulness-based training to teach basic meditations and related contemplative practices including standard Buddhist meditation practices including the four Immeasurables (compassion, equanimity, and empathic joy, and equanimity) (*n* = 69); (2) psychoeducational program to teach caregivers how to recognize and reduce workplace stress and educate caregivers how to meet the needs of individuals with intellectual disabilities and ASD (*n* = 67); and (3) in-service training-as-usual program which included education on behavior management, crisis intervention plans, emergency medications for severe aggressive behavior to self, peers, and staff, physical restraints, aversive contingencies and punishment strategies and skills training (*n* = 63). The study aimed to assess perceived stress, compassion satisfaction, compassion fatigue (i.e., burnout and secondary traumatic stress), and symptoms of depression after 32 weeks of implementation. Intervention adherence rates were high for the mindfulness program, psychoeducational program, and in-service training-as-usual at 95, 93 and 88%, respectively.

There were no significant differences among the three groups in compassion satisfaction levels at baseline. Compassion satisfaction increased significantly in the mindfulness program, followed by psychoeducation. No increase in compassion satisfaction was seen in the in-service training-as-usual condition. A mixed ANOVA indicated a moderate effect on caregivers’ compassion satisfaction. The mindfulness component of the Mindfulness-Based Positive Behavior Support (MBPBS) program was significantly better at enhancing compassion satisfaction and reducing caregiver-perceived psychological stress, burnout, secondary traumatic stress, and depression symptoms when compared to psychoeducation and in-service training as usual. Mindfulness training was the most effective of the three programs in enhancing caregiver quality of life.

In a qualitative interview study from Australia, O’Reilly (2017) described how paid carers used humor as a tool to provide compassionate care to youth with ABI who could not direct or perform their own care. The perspectives of youth with ABI receiving care were not sought. Humor was used to provide sensory stimulation (i.e., playfully tossing an item to the youth) and to respond to visual cues (i.e., tattoos and posters the youth have in their room). The paid carers’ interactions were not planned; they were specific to the youth with ABI and occurred naturally during the carers’ interactions with the youth. The carers perceived age-appropriate humor as an effective method of providing compassionate care. In one case, a paid carer recounted how a youth they were caring for responded to their humor:

“I suppose to know that you’re doing something positive in somebody’s life. You’re contributing something positive to someone’s life is really important and doing the best that you can with what you’ve got in front of you. You know with the care that you’ve got there, with the amount of communication that you do have, which for him is smiling and occasional sounds. So, when you get those it’s rewarding to your care and you know that maybe there’s a little bit more there, so you try to push on that a little bit.”

A qualitative study from the United States examined the family experience of critical care after pediatric TBI to develop a family-centered care model (Moore et al., 2015). One of the three major themes identified was “thorough, timely and compassionate communication.” The five factors associated with compassionate communication included: (1) thorough communication (i.e., involving parents in their child’s care plan, which indicated to parents that healthcare professionals were aware of their child’s needs); (2) use of multiple communication methods to ensure parents had a clear understanding of their child’s care; (3) timely communication when there was a status change or new critical information; (4) desire to be listened to by healthcare providers (i.e., parents highlighted that positive communication experiences were when providers engaged with the family and demonstrated empathy and respect for their experience); and (5) orienting parents on what to expect in critical care and post-discharge. These findings were used to develop a family-centered TBI care model which highlighted parent participation, frequent communication and coordinated care transitions, including continuity of information and maintenance of partnerships with families and care teams.

### Outcomes associated with compassion in the care of youth with childhood-onset disabilities

Self-compassion was measured using several outcome measures, including the Self-Compassion Scale Short Form (SCS-SF) (Robinson et al., 2018; Biddle et al., 2020) and the 26-item Self-Compassion Scale (Ivins-Lukse and Lee, 2021). Other studies used proxy outcome measures for compassion, the Parenting Goal Questionnaire (Conti, 2015), and the Professional Quality of Life rating scale (Singh et al., 2020). The Parenting Goal Questionnaires was developed specifically for use in Conti (2015) and were based on the compassionate and self-image goals questionnaire developed by Crocker and Canevello (2008). The Professional Quality of Life Scale is a 30-item scale which is used to measure compassion satisfaction, compassion fatigue, burnout and work satisfaction. The scale has a positive component [Compassion Satisfaction – “pleasure derived from being able to do one’s work well” (Singh et al., 2020)] and a negative component [Compassion Fatigue – “work-related, secondary exposure to extremely or traumatically stressful events” (Singh et al., 2020)]. Other quantitative outcomes evaluated included depression, stress, and life satisfaction.

Five studies explored self-compassion among parents of youth with childhood-onset disabilities (Conti, 2015; Robinson et al., 2018; Biddle et al., 2020; Ivins-Lukse and Lee, 2021; Iannuzzi et al., 2022) (Table 3). Overall, self-compassion among parents of youth with childhood-onset disabilities was associated with increased pride, quality of life and resiliency and lower levels of psychological distress, depression, shame, and guilt (Biddle et al., 2020).

**Table 3:** Intervention characteristics.

Study	Aims	Methods	Conceptualization	Use	Outcomes
Biddle et al. (2020)	To explore experiences of caregivers of children, adolescents and young adults diagnosed with FASD. Specifically the relationship between shame, guilt, pride, self-compassion and caregiver psychological distress.	Quantitative; cross-sectional survey	Self-compassion can be broadly described as the recognition and desire to alleviate one’s suffering with self-kindness and non-judgment, and has been shown to be associated with several positive outcomes (e.g., greater psychological health, reduced isolation, and improved wellbeing).		Caregiver psychological distress (DASS-21) was negatively correlated with self-compassion and pride, and positively correlated with shame and guilt. Caregivers with higher psychological distress had lower levels of self-compassion and pride, and higher levels of shame and guilt. There was a non-significant trend for self-compassion to be higher for non-biological caregivers (*M* = 3.16, SD = 0.69) compared with biological caregivers (*M* = 2.95, SD = 0.26) (M = 0.21, 95%CI [0.01 to 0.42], *t* (15.75) = 2.04, *p* = 0.059) Self-compassion and shame accounted for unique variance in psychological distress and pride and guilt did not. The findings suggest that self-compassion may represent a protective resource for caregivers, whereas shame may contribute to risk of distress.
Conti (2015)	To examine the relationships among compassionate and self-image parenting goals and feelings of satisfaction, efficacy and meaning among mothers of children with autism.	Quantitative; cross-sectional survey	Not defined	N/A	Compassionate parenting goals were more strongly endorsed by mothers of children with autism. Compassionate goal endorsement positively predicted family life satisfaction, parental self-satisfaction and having meaning in life. A positive relationship was observed between compassionate parenting goals and parenting efficacy. Compassionate parenting goals affect the instrumental dimension of mothers’ relationships with her children with autism and the affective dimension of these relationships. Adopting compassionate parenting goals may lead mothers to understand their children better and more confident in their parenting ability.
Iannuzzi et al. (2022)	To explore sibling and parents’ perspectives of youth with ASD	Qualitative; semi-structured interviews	Not defined	N/A	Experiences of having a sibling with ASD provided opportunity for developing values including acceptance, respect, and feelings of empathy and compassion. Neurotypical participants with siblings who had severe functional impairments and communication challenges expressed more compassion despite an increase in behavioural challenges. Parents of youth with severe functional impairments also expressed more compassion.
Ivins-Lukse and Lee (2021)	To explore the connections between self-compassion and stigma among parents of transition-age youth with IDDs	Quantitative; cross-sectional survey	Self-compassion represents a form of emotion-based coping, one that specifically involves how an individual relates to himself or herself when facing difficult experiences	N/A	Self-compassion was reported to partially mediate the relationship between courtesy stigma and caregiver depressive symptoms (effect = 0.28, CI [0.16, 0.45]). Self-compassion is negatively associated with depressive symptoms. Self-compassion could be a target point for interventions aimed at improving psychosocial adjustment among parents of transition-age youth with IDDs. Self-compassion can be a valuable coping strategy for parents of youth with IDD.
Moore et al. (2015)	To examine the family experience of critical care after pediatric TBI to develop a model of specific factors associated with family-centered care	Qualitative; semi-structured interviews	Not defined	Three major themes associated with high quality, FCC for families of children with TBI were identified: (1) thorough, timely and compassionate communication, (2) capacity building for families and providers, and (3) coordination of care transitions.	N/A
O’Reilly (2017)	To describe how paid carers use humor in providing compassionate post-intensive rehabilitation care to young adults with ABI who are unable to perform or direct their own care.	Qualitative; interviews	Not defined	Humor may be a rehabilitation tool to elicit responses from those with no verbal language.	N/A
Robinson et al. (2018)	To explore the construct of self-compassion in parents of young people adults with IDD	Quantitative; cross-sectional survey	Self-compassion is defined as being touched by and open to one’s own suffering, not avoiding or disconnecting from it, generating the desire to alleviate one’s suffering and to heal oneself with kindness,” self-compassion also involves “offering nonjudgmental understanding to one’s pain, inadequacies and failures, so that one’s experience is seen as part of the larger human experience”	N/A	A wide range of self-compassion levels were reported and were not associated with any specific parent demographics. Increased levels of self-compassion were related to lower levels of stress and depression. Self-compassion may offer resiliency against parenting challenges.
Singh et al. (2020)	To evaluate the comparative effects of three programs—mindfulness program, psycho-educational program, and in-service training-as-usual—on the quality of life of paid caregivers who provide services to adolescent and adult clients with ID and ASD	Quantitative; three-arm cluster RCT with two active conditions and a control condition	Not defined	The effects of the training on the caregivers’ quality of life were assessed in terms of perceived stress, compassion satisfaction, compassion fatigue (i.e., burnout, secondary traumatic stress), and symptoms of depression at the end of 32 weeks of implementation.	There were no significant differences among the three groups in compassion-satisfaction levels at base-line. Compassion satisfaction increased significantly in the mindfulness condition, followed by psychoeducation, but not in the in-service training-as-usual condition. Mixed ANOVA indicated a moderate effect on caregivers’ compassion satisfaction. Mindfulness component of the MBPBS program was significantly better at enhancing compassion satisfaction when compared to psychoeducation and in-service training-as-usual. Mindfulness training is the most effective of the three programs in enhancing caregiver quality of life.

ABI, acquired brain injury; ASD, autism spectrum disorder; DASS-21, Depression Anxiety Stress Scale; FCC, family-centered care; IDD, intellectual and developmental disabilities; MBPBS, Mindfulness-Based Positive Behavior Support; RCT, randomized controlled trial; TBI, traumatic brain injury.

In a cross-sectional study from Australia, caregivers of children diagnosed with FASD completed an online survey that included several standardized self-report measures for self-compassion, pride, guilt, shame, and psychological distress [i.e., SCS-SF, Depression Anxiety Stress Scale (DASS-21), and State Shame and Guilt Scale (SSGS)] (Biddle et al., 2020). Caregivers were defined as anyone who provided care for a youth with FASD, including adoptive parents (*n* = 97; 55.4%), foster parents (*n* = 46; 26.3%), grandparents (*n* = 14; 8%), biological parents (*n* = 10, 5.7%), aunts/uncles (*n* = 6, 3.4%), and step-parents (*n* = 2; 1.2%). Caregivers with high levels of self-compassion reported less psychological distress, shame and guilt (Biddle et al., 2020). Self-compassion was suggested to be a protective resource for caregivers, decreasing the risk of psychological distress and enhancing the caregiver’s quality of life. Biological caregivers reported increased guilt and a trend for lower self-compassion (non-significant) than non-biological caregivers. Biological and non-biological caregiver groups reported similar levels of psychological distress and shame.

Conti (2015) examined the relationships among compassionate and self-image parenting goals (i.e., concerned with others’ opinions of their child’s behavior) and feelings of satisfaction, efficacy and meaning in life among mothers of children with autism. Compassionate parenting goals aim to “gain a view of the world from the child’s perspective, show the child that his or her interests and abilities are truly valued, and tailor parenting efforts in a way that recognizes the child’s individual needs” (Conti, 2015). This approach allows the parent to sense the child’s experience and attempt to alleviate their distress or suffering. Compassionate parenting goals were shown to predict positive parenting outcomes, including family and parenting satisfaction, parenting efficacy, and meaning in life. Further, compassionate parenting goals were strongly endorsed by mothers of children with autism (Conti, 2015).

Iannuzzi et al. (2022) explored the lived experiences of neurotypical siblings of youth with ASD from the perspective of the neurotypical sibling as well as their parents. Siblings of youth with ASD completed a semi-structured interview and reported on their own experiences of having a sibling with ASD. Parents were asked to describe the experiences of their neurotypical child’s relationship with their sibling with ASD. Siblings of youth with ASD reported that they had many opportunities to learn and develop values, including feelings of compassion, empathy, acceptance, and respect from having a sibling with ASD. Further, participants with siblings who had significant functional impairments and communication challenges expressed a greater amount of compassion toward their siblings:

“I absolutely love my brother so much. I do think it’s different than other families. I think the fact that he’s nonverbal and has autism. I’ve had to make an effort to have a relationship more than one would with a neurotypical sibling. I felt like… it was a greater bond. [NT Sibling]”

Parents also shared similar observations about the relationship between siblings:

“[My NT daughter] knows how to identify with somebody and she will go over and make them feel comfortable… She finds things and pick up things that other people can’t. She’s got a huge sensitivity to feeling and making people feel like they fit in. [Parent]”

Siblings of youth with ASD reported having a level of compassion for others and expressing acceptance and gratitude for the challenges and rewards of having a sibling with ASD.

A cross-sectional survey study from the United States explored the connections between self-compassion and courtesy stigma [i.e., “being stigmatized by others due to their association with their child with an intellectual and developmental disability” (Ivins-Lukse and Lee, 2021)] among parents of transition-aged youth with intellectual and developmental disabilities (Ivins-Lukse and Lee, 2021). Self-compassion was found to be negatively associated with depressive symptoms. Further, self-compassion was found to partially mediate the relationship between courtesy stigma and depressive symptoms. This finding suggests that the association between stigma and caregiver well-being occurs in part because of the impact of courtesy stigma on parents’ self-compassion. The authors concluded that self-compassion was a valuable coping strategy and worthy of consideration as a target for interventions to improve psychosocial adjustment for parents of transition-age youth with intellectual and developmental disabilities.

Similar findings were reported in a quantitative cross-sectional study from Canada investigating the association between self-compassion and measures of well-being for parents of young people and adults with intellectual and developmental disabilities (Robinson et al., 2018). Increased levels of self-compassion were associated with lower levels of stress and depression. Further, increased levels of self-compassion were reported to offer resiliency against the challenges of parenting a child with intellectual and developmental disabilities, including self-criticism, isolation, and “rare opportunities” for self-care and mindfulness (Robinson et al., 2018). Self-compassion was important to well-being irrespective of parent stressors, including child diagnosis and parental burden.

## Discussion

### Summary of findings

This scoping review describes the conceptualization, use and outcomes associated with compassion in the care of youth with childhood-onset disabilities. Eight studies on compassion in the care of youth with childhood-onset disabilities were identified. Compassion was not defined *a priori* or posteriori in any of the included studies. Self-compassion was only conceptualized by established definitions *a priori* in three studies, specifically for parents or paid caregivers of youth with childhood-onset disabilities. Self-compassion among youth with childhood-onset disabilities was not discussed in any of the included studies.

Further, several studies liberally used the term ‘compassion’ and ‘compassionate care’ to describe a component of family-centered care (Moore et al., 2015) and clinical communication (O’Reilly, 2017). Qualitative studies from this review suggest that age-appropriate humor is a potential method of providing compassionate care. Additionally, mindfulness training was shown to increase self-compassion satisfaction. Parents who displayed self-compassion reported positive parenting outcomes, such as improved efficacy, satisfaction, pride, quality of life, and resilience. They also experienced lower levels of psychological distress, depression, shame, and guilt. Self-compassion was also found to be a coping mechanism and protective resource for caregivers, as it reduced the risk of psychological distress and improved their overall quality of life.

### Conceptualization of compassion

Compassion within the healthcare system has received global attention for many years with increased interest from clinicians, policymakers, patients and family members (Papadopoulos et al., 2016; Malenfant et al., 2022). While patients have often emphasized the importance of compassion to the overall quality of care they receive, there is limited evidence on how compassion is perceived by patients (Sinclair et al., 2016; Malenfant et al., 2022). Sinclair et al. (2016) conducted a scoping review on compassion within healthcare and identified gaps in the existing evidence base over 25 years (1988–2014). Seventy-five percent of articles selected for inclusion within Sinclair’s review were published within the last 5 years, highlighting the increasing interest in compassion within healthcare. Less than one-third of these studies focused specifically on patients, with the rest focusing on caregivers, clinicians and students (Sinclair et al., 2016). Interestingly, only a few studies investigated how patients defined compassion or assessed patient health outcomes or health-related quality of life (Sinclair et al., 2016). To bridge this gap, recent studies within the pediatric oncology literature have explored the conceptualization of compassion among children, their parents and healthcare providers, leading to the development of an empirical framework that depicts the dimensions of compassion for pediatric oncology patients (Sinclair et al., 2020, 2021, 2022).

However, the conceptualization of compassion or self-compassion according to youth with childhood-onset disabilities is not well-defined. Within this review, only three studies included a conceptualization of self-compassion from the perspective of parents and caregivers of youth with childhood-onset disabilities and used established definitions of self-compassion. None of the included studies provided a formal definition of compassion or discussed self-compassion among youth with childhood-onset disabilities.

### Use of compassion

Only two studies investigated the use of compassion among paid carers of youth with ABI, intellectual disability, and ASD (O’Reilly, 2017; Singh et al., 2020).

Using age-appropriate humor was described as a tool that may be used to provide compassionate care to individuals with an ABI (O’Reilly, 2017). Humor within the rehabilitation setting has become an area of interest for rehabilitation professionals and is considered to be an “optimal component of interpersonal experience,” helping to establish communication, diffuse tension, and assist rehabilitation professionals in building rapport and connection with patients (Taylor, 2020). Humor has also been shown to be a way to elicit responses from individuals with communication difficulties following TBI (Hyrkäs, 2005). Hyrkäs (2005) noted that paid carers who used “positive humor,” including laughing *with* care recipients and their peers, were found to motivate patients and improve their confidence in rehabilitation. Humor has also been shown to foster compassion by increasing feelings of belonging and understanding (Kfrerer et al., 2023), improving self-esteem, life satisfaction and optimism, which may lead to positive rehabilitation outcomes (Kfrerer, 2018; Schneider et al., 2018).

Additionally, thorough and timely communication was identified as another way to provide compassionate care, particularly in the acute care setting for young people with TBI (Moore et al., 2015). This involved informing parents regularly of any new or critical information and providing them with a detailed care plan for their child. Similarly, Sinclair et al. (2020) identified three facilitators of compassion within pediatric care, including communication, continuity of care, and coordination of care.

Communication encompasses the tone, style, language, and cultural sensitivity the health care provider uses to convey their message. Clear and direct discussions between healthcare providers and family members “promoted a holistic approach to pediatric care” (Sinclair et al., 2020). For instance, families of deceased children reported “profound distress” when news was delivered without compassion or in situations where healthcare providers behaved insensitively. An essential factor in building trust between healthcare providers and patients of critically ill youth was honest, inclusive, compassionate and coordinated communication (Sinclair et al., 2020). In a systematic review by Manikandan and colleagues (2021), patients with cerebral palsy who had a speech impairment found it difficult to communicate with service providers, having to rely on assistive devices or interpreters to access health services. Some studies in this review reported a lack of understanding and expertise in providing care for individuals with cerebral palsy who have complex communication needs. Several studies reported that healthcare workers often ignored individuals with cerebral palsy and spoke directly with their caregivers, and often used complex medical jargon due to this lack of understanding and expertise (Larivière-Bastien et al., 2013; Manikandan et al., 2022). These facilitators (continuity of care, communication, and coordination of care) are elements for providing compassionate care. Providing compassion training to healthcare providers which focuses on effective (i.e., active listening, building rapport) and affective (i.e., engaging with patients in a sensitive way, addressing emotional distress) communication skills (Sinclair et al., 2020) may be beneficial in improving the overall quality of care provided to youth with childhood-onset disabilities and their families.

Further, the mindfulness component of the MBPBS training program for paid caregivers significantly enhanced compassion satisfaction and reduced caregiver-perceived psychological stress, burnout, secondary traumatic stress, and depression symptoms compared to psychoeducation and in-service training-as-usual (Singh et al., 2020). This was the first study to explore the use of MBPBS to enhance compassion satisfaction and decrease compassion fatigue among paid caregivers of youth with childhood-onset disabilities. These findings support the effectiveness of MBPBS in reducing perceived psychological stress and staff turnover among paid caregivers. Staff in a congregate care facility trained in MBPBS reported a greater reduction in psychological stress than staff who received standard training (Singh et al., 2016). Similarly, group home staff who received intensive MBPBS training for 7 days reported reduced levels of perceived stress and zero staff turnover (Singh et al., 2015). A meta-analysis of mindfulness-based interventions on self-compassion among healthcare professionals found that mindfulness-based interventions implemented at an organizational level create a supportive and compassionate environment, leading to improvements in self-compassion, stress, depression and anxiety (Wasson et al., 2020). Improving compassion among healthcare providers can, in turn, lead to improved outcomes for youth with childhood-onset disabilities by alleviating the suffering of patients, improving their overall well-being, and enhancing the quality of care they receive from healthcare providers (Sinclair et al., 2016, 2017; Singh et al., 2020).

Several scoping reviews on compassion within the healthcare context have noted that compassion is often studied in healthcare providers more frequently than in patients (Sinclair et al., 2016; Malenfant et al., 2022; Simpson et al., 2022). However, within the childhood-onset disability population, none of the reviewed studies described the interpersonal qualities or skills that define compassionate pediatric healthcare providers. Similarly, in a scoping review conducted by Sinclair et al. (2020) on the key factors, facilitators and barriers of compassion in pediatric healthcare, the authors noted that the included studies also did not identify the behaviors, qualities or skills that healthcare providers should possess to provide compassion in a clinical setting. Future studies should seek to develop a foundational understanding of compassion within the childhood-onset disability population and understand how healthcare providers can use compassion in caring for youth with childhood-onset disabilities and their families.

### Outcomes associated with compassion

Compassionate parenting goals were shown to predict positive parenting outcomes, including family and parenting satisfaction, parenting efficacy, and meaning in life (Conti, 2015). Further, compassionate parenting goals were strongly endorsed by mothers of children with autism (Conti, 2015). Curley and Kotera (2023) conducted a qualitative study on the perceptions of compassionate parenting in parents of youth with autism spectrum disorder (Curley and Kotera, 2023). Compassionate parenting improved the parent’s quality of life and reduced their stress by helping them de-escalate stressful parenting situations (Curley and Kotera, 2023). Further, compassionate parenting provides an opportunity for youth to model compassion, which is important for a youth’s psychosocial development (Curley and Kotera, 2023). Parents also noted that compassion was an important part of their parenting to communicate a sense of worth, love and understanding with their child (Curley and Kotera, 2023).

Self-compassion in parents of youth with childhood-onset disabilities was the most common outcome measure. Self-compassion among parents is associated with increased quality of life and resiliency and lower levels of psychological distress, depression, shame, and guilt. There is a well-established link between self-compassion and well-being in parents of youth with intellectual and developmental disabilities (Benn et al., 2012; Bazzano et al., 2015; Neff and Faso, 2015; Wong et al., 2016). Positive dimensions of self-compassion were found to buffer the parental quality of life and the child’s behavior and symptoms. This is supported by previous research that self-compassion can predict psychological outcomes in parents of youth with ASD (Wong et al., 2016). As self-compassion is a modifiable trait, future interventions should target self-compassion to improve quality of life and reduce parenting stress (Benn et al., 2012). Such interventions may improve child outcomes (Osborne et al., 2008; Osborne and Reed, 2009; Dabrowska and Pisula, 2010; Bekhet et al., 2012), and depression and anxiety among parents (Bitsika and Sharpley, 2004; Bitsika et al., 2013).

Sinclair et al. (2020) also noted that many studies discussed compassion in a “subsidiary fashion,” and similarly, within this review, compassion was often included as one of several outcome measures and not the focus of the study (Moore et al., 2015; O’Reilly, 2017; Iannuzzi et al., 2022). Additional research on the outcomes of compassion and self-compassion among youth with childhood-onset disabilities using measures specifically validated for this population is required. To improve patient outcomes and better address the needs of young individuals with childhood-onset disabilities, it is essential to investigate how compassion and self-compassion are understood by caregivers and particularly the youth themselves. Future studies can employ a grounded theory approach, as previously used in palliative care settings (Sinclair et al., 2017, 2018), to develop a patient-, caregiver-, and healthcare provider-informed clinical framework. This approach can help establish an empirically-derived theoretical model of compassion specifically for young individuals with childhood-onset disabilities. Such insights can pave the way for better care and support for this population.

### Strengths and limitations

This scoping review provides an in-depth summary of the evidence for the conceptualization, use and outcomes associated with compassion in caring for youth with childhood-onset disabilities. This review also highlights gaps in the evidence base, including the paucity of primary studies investigating compassion from the perspective of youth with childhood-onset disabilities. Studies that included compassion-related concepts, including empathy and sympathy, were only considered in this review if they were discussed in conjunction with compassion. While the exclusion of sympathy and empathy from the review may be seen as a limitation, Sinclair et al. (2016) note that sympathy and empathy are often conflated when discussing compassion. These concepts have distinct characteristics, outcomes and responses (Sinclair et al., 2017). Further, while several validated measures of compassion were used (26-item Self-Compassion Scale; Self-Compassion Scale Short Form), these measures have yet to be validated within the current study populations.

Additionally, the generalizability of the review findings is limited as only studies published in English were selected for inclusion, and six of the eight included studies were conducted in North America. Previous studies have noted that there are cultural variances in the conceptualization and expression of compassion globally (Schantz, 2007; Goetz et al., 2010; Chrousos, 2014; Papadopoulos et al., 2016, 2017; Singh et al., 2020; Malenfant et al., 2022). Cross-cultural studies on compassion are needed to investigate these differences further. As well, most study participants within the included studies identified as “Caucasian.” Additional studies with ethnically diverse populations are needed to understand how different ethnic groups experience and conceptualize compassion and how healthcare providers can provide compassion when caring for patients of various ethnic backgrounds (Frampton et al., 2013; Babaei et al., 2016; Singh et al., 2018).

## Conclusion

There is limited literature on the conceptualization, use, and outcomes of compassion in the care of youth with childhood-onset disabilities. Compassion was not defined *a priori* or *a posteriori* in any of the included studies. Self-compassion among youth with childhood-onset disabilities was not discussed in any of the included studies. Self-compassion may be an effective internal coping process among parents of youth with childhood-onset disabilities. Parents who displayed self-compassion reported positive parenting outcomes, such as improved efficacy, satisfaction, pride, quality of life, and resilience. Further, several studies liberally used the terms ‘compassion’ and ‘compassionate care’. Literature on the use of compassion by healthcare providers treating youth with childhood-onset disabilities is also limited. Future research is required to understand the conceptualization of compassion from the perspective of youth with childhood-onset disabilities and their caregivers.

## Data availability statement

The original contributions presented in the study are included in the article/Supplementary material, further inquiries can be directed to the corresponding author.

## Author contributions

EP: Conceptualization, Data curation, Formal analysis, Methodology, Project administration, Writing – original draft, Writing – review & editing. SP: Data curation, Writing – original draft, Writing – review & editing. RS: Conceptualization, Methodology, Writing – original draft, Writing – review & editing. MN: Conceptualization, Methodology, Writing – original draft, Writing – review & editing. MP: Conceptualization, Methodology, Writing – original draft, Writing – review & editing. LP: Methodology, Writing – original draft, Writing – review & editing. MB: Conceptualization, Methodology, Supervision, Writing – original draft, Writing – review & editing. SM: Conceptualization, Formal analysis, Funding acquisition, Methodology, Supervision, Writing – original draft, Writing – review & editing.
