# Supplementary material for: Conceptualization, use, and outcomes associated with compassion in the care of youth with childhood-onset disabilities: a scoping review
Source: Front Psychol. 2024 Jun 7;15:1365205. doi: 10.3389/fpsyg.2024.1365205 (PMC11192198; doi:10.3389/fpsyg.2024.1365205)
Supplement: Supplementary file 2 [file Table_2.DOCX]

**Appendix S2**

**MEDLINE Search Strategy**

Database: Ovid MEDLINE: Epub Ahead of Print, In-Process & Other Non-Indexed Citations, Ovid MEDLINE® Daily and Ovid MEDLINE® <1946-Present>

Search Strategy:

--------------------------------------------------------------------------------

1 (compassion$ adj5 "patient care").tw.

2 (compassion$ adj5 care).tw.

3 (compassion$ adj5 patient$).tw.

4 (compassion$ adj5 outcome$).tw.

5 (empath$ adj5 "patient care").tw.

6 (empath$ adj5 care).tw.

7 (empath$ adj5 patient$).tw.

8 (empath$ adj5 outcome$).tw.

9 Empathy/

10 or/1-9

11 Adolescent/

12 Young Adult/

13 pediatric?.tw.

14 paediatric?.tw.

15 adolescent?.tw.

16 adolescence.tw.

17 teen?.tw.

18 teenage.tw.

19 teenager?.tw.

20 juvenile.tw.

21 youth.tw.

22 (young adj person?).tw.

23 (young adj people).tw.

24 (young adj adult?).tw.

25 (young adj adulthood).tw.

26 (young adj men).tw.

27 (young adj women).tw.

28 (young adj male?).tw.

29 (young adj female?).tw.

30 (emerging adj adult$).tw.

31 Adolescent Medicine/

32 Adolescent Health Services/

33 Hospitals, Pediatric/

34 or/11-33

35 Cerebral Palsy/ [ childhood onset disabilities ]

36 (cerebral adj pals$).tw.

37 exp Spinal Dysraphism/

38 (spina adj bifida).tw.

39 Meningomyelocele/

40 Meningocele/

41 myelomeningocele.tw.

42 meningocele.tw.

43 (spinal adj dysraph$).tw.

44 Brain Injuries/

45 Hematoma/

46 exp Meningitis/

47 exp Encephalitis/

48 encephalitis.tw.

49 meningitis.tw.

50 (brain adj2 injur$).tw.

51 (head adj2 injur$).tw.

52 exp Disabled Persons/

53 disabilit$.tw.

54 disabled.tw.

55 handicap$.tw.

56 (physical adj2 impair$).tw.

57 (impaired adj person$).tw.

58 (impaired adj patient$).tw.

59 (limb adj deformit$).tw.

60 (limb adj deformat$).tw.

61 (limb adj defect$).tw.

62 amputee$.tw.

63 amputation$.tw.

64 Musculoskeletal Diseases/

65 (muscle adj disease$).tw.

66 musculoskeletal.tw.

67 (musculo adj skeletal).tw.

68 exp Achondroplasia/

69 achondroplasia.tw.

70 Osteogenesis Imperfecta/

71 (osteogenesis adj imperfecta).tw.

72 Marfan Syndrome/

73 marfan$.tw.

74 (spina adj bifida$).tw.

75 exp Muscular Dystrophies/

76 (muscular adj dystroph$).tw.

77 exp Muscular Atrophy/

78 (muscular adj atroph$).tw.

79 myopathy.tw.

80 myopathies.tw.

81 Myositis/

82 myositi$.tw.

83 polymyositi$.tw.

84 Polymyositis/

85 exp Dystonia/

86 dystonia.tw.

87 (back adj pain$).tw.

88 Back Pain/

89 (neck adj pain$).tw.

90 Neck Pain/

91 (shoulder adj pain$).tw.

92 Shoulder Pain/

93 fibromyalgia.tw.

94 Fibromyalgia/

95 osteoarthr$.tw.

96 exp Osteoarthritis/

97 arthritis.tw.

98 arthritides.tw.

99 polyarthriti$.tw.

100 "inflammatory joint disease$".tw.

101 bechterew$.tw.

102 Spondylitis, Ankylosing/

103 spondyl$.tw.

104 dysmeli$.tw.

105 Myasthenia Gravis/

106 myasthenia.tw.

107 exp Paralysis/

108 parapleg$.tw.

109 Paraplegia/

110 hemipleg$.tw.

111 Hemiplegia/

112 tetrapleg$.tw.

113 Quadriplegia/

114 (cerebellar adj ataxia$).tw.

115 Cerebellar Ataxia/

116 "traumatic brain injur$".tw.

117 "spinal cord injur$".tw.

118 (ehlers adj danlos).tw.

119 Ehlers-Danlos Syndrome/

120 Dyskinesias/

121 Chorea/

122 chorea.tw.

123 exp Hydrocephalus/

124 hydrocephalus.tw.

125 exp Epilepsy/

126 seizure$.tw.

127 epilep$.tw.

128 exp Neuromuscular Diseases/

129 (guillain adj barre$).tw.

130 polyneuropath$.tw.

131 neuropath$.tw.

132 exp Cerebrovascular Disorders/

133 Stroke/

134 stroke.tw.

135 cerebrovasc$.tw.

136 (cerebral adj vasc$).tw.

137 Attention Deficit Disorder with Hyperactivity/

138 ADHD.tw.

139 (attention$ adj3 disorder$).tw.

140 (attention adj2 defic$).tw.

141 Deafness/

142 Hearing Loss/

143 deaf$.tw.

144 (hearing adj impair$).tw.

145 (hearing adj disorder$).tw.

146 (hearing adj loss$).tw.

147 Mental Retardation, X-Linked/

148 Intellectual Disability/

149 Down Syndrome/

150 Fragile X Syndrome/

151 Fetal Alcohol Spectrum Disorders/

152 Angelman Syndrome/

153 (mental$ adj retard$).tw.

154 (intellectual$ adj2 disab$).tw.

155 (intellectual$ adj2 impair$).tw.

156 (down$ adj syndrome).tw.

157 (fragile adj x).tw.

158 (fetal adj alcohol).tw.

159 (angelman adj syndrome).tw.

160 Learning Disorders/

161 Developmental Disabilities/

162 (learning adj disorder$).tw.

163 (learning adj disab$).tw.

164 (development$ adj disab$).tw.

165 (development$ adj disorder$).tw.

166 Motor Skills Disorders/

167 Movement Disorders/

168 (motor adj2 disorder$).tw.

169 (movement adj2 disorder$).tw.

170 "developmental coordination disorder$".tw.

171 Communication Disorders/

172 Language Disorders/

173 Speech Sound Disorder/

174 Social Communication Disorder/

175 Childhood-Onset Fluency Disorder/

176 (communication$ adj disorder$).tw.

177 (language adj disorder$).tw.

178 (speech adj2 disorder$).tw.

179 (fluency adj disorder$).tw.

180 Child Development Disorders, Pervasive/

181 Autism Spectrum Disorder/

182 Asperger Syndrome/

183 Autistic Disorder/

184 Rett Syndrome/

185 autis$.tw.

186 asperger$.tw.

187 rett$.tw.

188 "childhood disintegrative disorder$".tw.

189 (heller$ adj syndrome).tw.

190 (disintegrative adj psychosis).tw.

191 Child Behavior Disorders/

192 Conduct Disorder/

193 "Attention Deficit and Disruptive Behavior Disorders"/

194 (behavio?r adj disorder$).tw.

195 (conduct adj disorder$).tw.

196 Tics/

197 Tic Disorders/

198 Tourette Syndrome/

199 Stereotypic Movement Disorder/

200 tic$.tw.

201 tourette$.tw.

202 Blindness/

203 Vision, Low/

204 blind$.tw.

205 (low adj2 vision).tw.

206 (visual$ adj impair$).tw.

207 or/35-206

208 10 and 34 and 207

209 exp Animals/ not (exp Animals/ and Humans/)

210 208 not 209
