# Supplementary material for: Conceptualization, use, and outcomes associated with compassion in the care of youth with childhood-onset disabilities: a scoping review
Source: Front Psychol. 2024 Jun 7;15:1365205. doi: 10.3389/fpsyg.2024.1365205 (PMC11192198; doi:10.3389/fpsyg.2024.1365205)
Supplement: Supplementary file 3 [file Table_3.DOCX]

## **Appendix S3**

## **PRISMA Flow Diagram**

**Identification of studies via databases and registers**

Duplicate records removed

(***n* = 895)**

Records identified through searching multiple databases

**(*n* = 2048)**

**Identification**

Records excluded

**(*n* = 945)**

Full-text articles not found **(*n* = 2)**

Records screened after duplicates removed

**(*n* = 1153)**

**Screening**

Full-text articles excluded **(*n* =198)**

No primary data on compassion **(*n* = 147)**

Wrong study design **(*n* = 34)**

Adult population (mean age of youth > 26 years) **(*n* = 11)**

Wrong language **(*n* = 6)**

Full-text articles assessed for eligibility

**(*n* = 206)**

Full-text articles included

**(*n* = 8)**

**Included**
